# Repentance as Rebuke: Betrayal and Moral Injury in Safety Engineering

**DOI:** 10.1007/s11948-022-00412-2

**Published:** 2022-11-14

**Authors:** Sidney W. A. Dekker, Mark D. Layson, David D. Woods

**Affiliations:** 1 Safety Science Innovation Lab, HLSS, Griffith University, Macrossan Building N16, Room 2.43, 170 Kessels Road, Nathan Campus, Brisbane, QLD 4111 Australia; 2 Delft University, Delft, The Netherlands; 3 St Mark’s National Theological Centre, School of Theology, Charles Sturt University, Canberra Campus, Australia; 4 Department of Integrated Systems Engineering, The Ohio State University, Columbus, OH USA

**Keywords:** Moral injury, Betrayal, Engineering ethics, Boeing 737MAX, Federal Aviation Administration, Accident

## Abstract

Following other contributions about the MAX accidents to this journal, this paper explores the role of betrayal and moral injury in safety engineering related to the U.S. federal regulator’s role in approving the Boeing 737MAX—a plane involved in two crashes that together killed 346 people. It discusses the tension between humility and hubris when engineers are faced with complex systems that create ambiguity, uncertain judgements, and equivocal test results from unstructured situations. It considers the relationship between moral injury, principled outrage and rebuke when the technology ends up involved in disasters. It examines the corporate backdrop against which calls for enhanced employee voice are typically made, and argues that when engineers need to rely on various protections and moral inducements to ‘speak up,’ then the ethical essence of engineering—skepticism, testing, checking, and questioning—has already failed.

## The Repentance

Not long ago in this journal, Herkert et al. (2020) analyzed and commented on the Boeing 737 MAX accidents and their ethical implications for engineering. One of the key calls is the fostering of moral courage: the courage to act on one’s moral convictions including adherence to codes of ethics, and strengthening the voice of engineers within large organizations. At the end of their article, Herkert and colleagues intimate a sense of *déjâ vu*—large-scale tragedies involving engineering decision-making are not new, nor are the calls for engineers to speak up and for their organizations to empower them (Feldman, 2004; Vaughan, 2005; Verhezen, 2010). Even more recently in this journal, Englehardt, Werhane and Newton pick up on this and point to the immense difficulty and ethical challenges for engineers in the face of organizational malfeasance (2021). We agree with Englehardt and colleagues: if an engineer needs ‘moral courage’ simply to do their job, then there is a major moral and systemic (or, as Herkert and colleagues say: ‘macroethical’) failure whose remedies cannot sustainably or reliably come from front-line moral heroism (cf. Tkakic, 2019). In this paper, we take this a step further, tracing the repentance and rebuke of one particular safety engineer (employed by the regulator, the Federal Aviation Administration) and explore what they mean for the question of moral courage in engineering.

Early in 2021, a Federal Aviation Administration (FAA) safety engineer based in Seattle, publicly denounced the federal regulator’s role in approving the Boeing 737MAX—a plane involved in two crashes that together killed 346 people. What drove him to speak out while still employed was his renewed faith, deeply intertwined with an active repentance for his role in failing to prevent the disasters (Gates, 2021). In a detailed letter sent to one of the families involved in the second crash, and several interviews, the safety engineer explained how he should have been, but was not, among the regulator’s specialists who assessed the MAX’s critical new flight control software. The safety engineer had no involvement until after the first MAX accident in Indonesia in 2018. Senior FAA managers had focused on fulfilling the demands of industry, while itself struggling to meet its regulatory mandate under conditions of deregulation and resource limitations (Englehardt et al., 2021). As a result, manufacturers did much of their own certification, a process that could leave senior engineers and unaware or ignorant of critical new systems or changes and additions to existing systems. Boeing had felt under pressure its employees to keep the implications of design and software changes to the 737 minor or invisible, and get it through regulatory approval without extra pilot training requirements (Defazio & Larsen, 2020).

While the safety engineer’s exclusion may have acted to mitigate feelings of remorse and guilt, he was the most experienced engineer in the regulator’s Seattle office. He admitted afterward that he “felt a strong conviction that I should help with healing the families of the 737 MAX crashes” (Gates, 2021, p. 13). He indicated how his regret arose from his failure to deploy the responsibilities of his role and expert knowledge. Sharpe (2004) reminds us how this would not so much be about exacting retrospective responsibility, about ‘backward-looking accountability’ which focuses on finding someone to blame for (not) having done something. Rather, it is a reminder of forward-looking accountability, linked to goal-setting and moral deliberation which themselves are embedded in particular roles that people occupy. Prospective responsibility, or forward-looking accountability, is oriented toward deliberative and practical processes involved in setting and meeting goals. In the case of safety regulation, the overriding goal of the entire system is avoiding harm to other people. A failure of forward-looking accountability, then, can be seen here as not deploying the system’s role or knowledge in the service of risk reduction and harm avoidance.

## The Moral Injury

‘Moral injury’ is increasingly recognized as one consequence when one’s ethical framework is broadsided or even broken by the actions of others or oneself. The term was first used by Shay in the mid 1990s to describe the “betrayal of what is right by someone in legitimate authority in a high stakes situation” (Shay, 2014, p. 181). Moral injury can be triggered by a self-accusation that is caused by something one did or failed to do. But things can also be done to someone, who suffers moral injury as a result. This means that a betrayal by those in authority within manufacturer or regulator would have been but one aspect of the moral injury. Moral injury also arises when one transgresses one’s own moral compass—leaving what Litz et al. (2009, p. 697) describe as “the lasting psychological, biological, spiritual, behavioral and social impact of perpetrating, failing to prevent, or bearing witness to acts that transgress deeply held moral beliefs and expectations.” The safety engineer’s would in that case have been a double moral wound.

Betrayal is a central theme for those morally injured and has been an almost ubiquitous human experience over the ages. Individuals such as Judas, Brutus, and Cassius betray leaders and nations, leading Dante to envision their destiny as the very center circle of hell with all other betrayers. These historical betrayals have often focused on the individual as the traitor who breaks the fraternal bonds of ‘thick’ human relationships (Margalit, 2017). Organizations can betray individuals, with destructive consequences (Smith & Freyd, 2014). Smith and Freyd focused on organizations that betray individuals who report sexual abuse, but others notice institutional betrayal in cases of public safety, finding that distrust and a sense of betrayal among people gets heightened when the disaster or crisis can be traced to human agency, to human-made decisions, judgments or errors (Sobeck et al., 2020). When an engineer believes they are part of an organization dedicated to safety in a high stakes context such as aviation, only to find this dedication to safety is getting—or has been—undermined, the concept of institutional betrayal must be considered. Roger Boisjoly’s failed attempts to stop the Challenger launch decision in 1986 is a well-publicized precedent (Vaughan, 1996).

Hindsight and outcome knowledge of course tend to single out the possible role of one’s omission(s). They connect the bad outcome to one’s inaction in a way that (over-)emphasizes one’s role and tends to finesse the contribution of a host of other factors (Baron & Hershey, 1988). Many of those factors would likely have overridden whatever one might have done in an attempt to alter the organizational course of action (Dekker et al., 2011). Memory of such events, however, are vividly organized around the counterfactual—‘what if I had acted differently at that juncture or opportunity?’ (Dekel & Bonanno, 2013; Fischhoff & Beyth, 1975). It implies that repentance is called for because it was a supposedly rational choice to not do the right thing (Dekker, 2007).

## The Humility and Hubris

Which, of course, it wasn’t. Technologies, says Wynne, are ‘unruly.’ They are less orderly, less rule-bound, less controlled and less universally reliable than we think. Technology evolves uncertainly according to innumerable ad hoc judgments and assumptions. It may present in a neat aesthetic of tidy practices and compliant regulatory submissions and formal procedures, but engineers, Wynne reminds us, are operating with far greater levels of ambiguity; they need to make judgements in less than clearly structured situations. This is particularly the case when a completed system is seen as no more than the sum of its parts (indeed, a version of the ‘many hands’ problem noted by Herkert et al.) which is not going to be changed in its essence because of some minor aerodynamic or software tweaks. This ignores the complex interactions and possible emergence of novel behaviors that weren’t in the original equation set (the aerodynamic pitch-up problem that eventually led Boeing to put in MCAS is itself an example of that). Eventually only the technological system, unleashed into practice, becomes the ‘real’ test of its own reliability and safety (Weingart, 1991). This realization, and the necessary humility in the face of complexity and uncertainty, demands at least in theory an entirely different response:

After the unthinkable has happened — ‘Why did you ever think there was such a thing as zero risk, and how naive can you be to imagine that technical knowledge does not harbour areas of uncertainty, even legitimate ignorance? We cannot be blamed for the inevitable uncertainties that exist in developing expert knowledge and technological systems’. (Wynne, 1988, p. 150)

But that may not be typical for how an engineer feels about her or his contribution to the success and failure of a technology. An engineer may be forgiven for—and indeed be professionally committed to—trying to reduce the uncertainties inherent in a technology and its deployment. For this, engineering encompasses a wide range of activities that, at their core, are tradeoffs between numerous design goals, under conditions of limited means, knowledge and technological capability, in order to achieve particular goals. Gaining experience with a legacy system, and making seemingly ‘minor’ tweaks to it, can actively contribute to a sense of certainty that risks are both known and under control. It is ironically a lack of failure—at least in recent history—with the system in question that can help set it on course for failure (Petroski, 2000). The greatest risk, indeed, of already safe but complex systems lies in their apparent absence of risk: their continued success (Dekker, 2011). As Petroski explains:

Success and failure in design are intertwined. Though a focus on failure can lead to success, too great a reliance on successful precedents can lead to failure. Success is not simply the absence of failure; it also masks potential modes of failure. Emulating success may be efficacious in the short term, but such behavior invariably and surprisingly leads to failure itself. (Petroski, 2018, p. 3)

Petroski warns how a strategy of emulating one’s own past success is braided through the human tendency towards hubris about safety (Petroski, 2006). As later authors summed it up: “despite the best intentions of all involved, the objective of safely operating technological systems could be subverted by some very familiar and ‘normal’ processes of organizational life” (Pidgeon & O’Leary, 2000, p. 16). One of these is to assure a market for the technology developed, which involved an early commitment to maximum interoperability of the various generations of 737s—particularly to avoid the imposition of additional pilot training requirements by the regulator. Control software modifications were necessary to achieve that. They seemed only small, meant to yield local advantages in similarity, efficiency or versatility (Woods & Dekker, 2000).

Toward the FAA, this meant that nothing noteworthy would or should be revealed outside the narrow scope of the earliest tests—which helped cement an addition to the control system as only ‘hazardous.’ This in turn meant that limited (or no further) testing was required, because earlier tests had shown that no tests were required (a engineering circularity that has been referred to as ‘the self-licking ice cream cone’ (Worden, 1992)). But in the meantime, in order to handle new aerodynamic discoveries, the manufacturer had to enhance the automatic control system’s power, with limited disclosure to the regulator and other stakeholders (even internal ones) (FAA, 2020).

What the reforms proposed by the FAA in the wake of the MAX accidents don’t do assertively—as more technology and automation will surely penetrate legacy systems and form the basis of novel ones—is acknowledge that tests must have the potential to reveal factors that fall outside of what engineering thought needed to be considered to that point. Testing has to be able to reveal things that can matter, things which engineers didn’t know would come up, didn’t think needed to be considered, or assumed would not have mattered even if they did. There is no (systems) engineering unless it actualizes a capability to recognize anomalies that lead to re-assessments and re-designs. The risk that engineering will uncover problems and require change and further testing has to be accepted as part of engineering and engineering oversight—despite budgetary and timeline implications. In the face of increasingly complex systems, regulatory work runs the risk of being piecemeal and narrow and may not be able to uncover bigger gaps or orphaned concerns. The regulator specifically has to answer the question whether engineering has sufficiently questioned or re-assessed the proposed designs and remain vigilant for other pressures that undercut or eliminate engineering’s role to question and test its own work.

## The Decay and the Disaster

Such pressures, however, were rife—even within the FAA. The engineer’s repentance comes not from a failure of one employee to muster the heroism to ‘voice’ his concerns. Instead, the entire system of prospective accountability on which a safety regulator’s work is supposedly founded, had eroded due to a number of factors. A US Senate inquiry in the aftermath of the MAX accidents found:

common themes among the allegations including insufficient training, improper certification, FAA management acting favorably toward operators, and management undermining of frontline inspectors. The investigation revealed that these trends were often accompanied by retaliation against those who report safety violations and a lack of effective oversight, resulting in a failed FAA safety management culture. (Wicker, 2020, p. 2)

The ‘failed safety management culture,’ of a regulator was itself a complex result of forces that had been decades in the making. Deregulation and cutbacks at some point leave a government agency with few options but to rely on the very manufacturer they are supposed to regulate for technical know-how and expertise (Bier et al., 2003). The resulting safety sacrifices are predictable; they are the systemic effects of deliberate political choices related to deregulation. Erosion of the organization’s core functions continues even while people inside cling to the aesthetics of its former authority and an idealized image of its own character. The most important consequence of such

decay is a condition of generalized and systemic ineffectiveness. It develops when an organization shifts its activities from coping with reality to presenting a dramatization of its own ideal character. In [such an] organization, flawed decision making of the sort that leads to disaster is normal activity, not an aberration. (Schwartz, 1989, p. 319)

By the time the MAX was assessed and certified, the safety engineer described the FAA as an organization with a militaristic chain of command, in which lower-level employees could offer opinions when asked but otherwise must ‘sit down and shut up.’ Which, the engineer now says, he wished he hadn’t (Gates, 2021). After the second crash of a Boeing 737MAX, the engineer still wasn’t assigned to work on the fix, so he attended meetings on his own accord and offered his expertise. A program manager asked him why he was there, and the engineer answered “because I’m pissed” (Gates, 2021, p. 13). Such repentant outrage at having been betrayed and excluded, and “restrained by reason and a resolve to see justice” (Fox, 2019, p. 211), can serve as a rebuke only to those who are attentive and receptive.

## The Remedies

One popular remedy (or putative way to pre-empt the very situation that might lead to moral injury) is telling engineers to try harder: the requirement or expectation on engineers and knowledgeable others to ‘speak up;’ to use their voice even in the face of pressures to remain silent. ‘Employee (or engineer) voice’ in this sense is known in the literature as:

any attempt at all to change, rather than to escape from, an objectionable state of affairs, whether through individual or collective petition to the management directly in charge, through appeal to a higher authority with the intention of forcing a change in management, or through various types of actions or protests, including those that are meant to mobilize public opinion. (Hirschman, 1970, p. 30)

Margalit (2017) recognizes the possibility of retaliation. Insiders who speak up against the express (or tacit but dominant) goals of their organization can be portrayed as a traitor to their employer, community and colleagues. Speaking up can be a relationally fraught exercise, regardless of legal protections or professional exhortations. So in order to do it,

…engineers need support. They cannot be expected to sacrifice their jobs and perhaps their careers by standing up to management power. In addition to government whistle-blowing protection, professional engineering associations need to provide legal, financial and employment services to individuals who are unfairly punished for speaking out on unsafe engineering projects. This is an example of ‘countervailing powers’ … needed in organizations working in high-risk environments. (Feldman, 2004, p. 714)

The FAA should have been the right place for this, which should perhaps have gone without saying. Not only is the very role of a regulator the discharge of its prospective accountability (and hearing bad news from employees is very much part of that), organization science suggests speaking up in government organizations should actually be easier. Research shows that:

employees who experienced psychological safety were more likely to exhibit voice behavior; employee voice, in turn, promoted work engagement. (Ge, 2020, p. 1)

Private enterprise doesn’t enjoy the best preconditions for such a climate of psychosocial safety (Becher & Dollard, 2016; Zadow et al., 2017) and employee engagement (Kaufman, 1960). Everything (and everyone) needs to be focused (in principle) on the company’s mission, and focused on short-term results (Galic et al., 2017). Middle managers in private business are more rule-bound and protocol-conscious and pay more deference to formal structure than their government counterparts. One explanation lies in the contingent nature of private employment and hierarchical control and accountability:

Business organizations clearly have a greater capacity to exert such control, and by implication to heighten individual sensitivity to the structural instruments of control. Such organizations may coerce compliance, if necessary up to the point of discharging an employee … this is a critical power. A middle manager in an economic organization is accordingly under constant competitive pressure to produce results and to display the norms and values prevailing in his administrative climate. Security, advancement, and ultimate success are conditioned on acceptable performance and behavior throughout the managerial career. (Buchanan, 1975, p. 436)

In government agencies, the relationship between individual work and organizational mission is less clear-cut. Government agencies also have stronger systems of appeal that acknowledges the procedural due process rights of individuals (whether employees or citizens). In government agencies, Buchanan (1975) found that control and accountability happen largely by persuasion rather than by control, surveillance, counting and coercion like in private corporations. The differences, however, are perhaps too easily overstated, and less clear-cut than they once were. There are complex interpersonal, social, cultural, emotional, hierarchical, ethical and operational factors that all enter into deliberations about speaking up or not speaking up (Orasanu & Martin, 1998; Salas et al., 2006; Weber et al., 2018). The coalescence of constraints and expectations across both government and private organizations since the 1980s has gradually erased some of the unique aspects of federal employment as offering more autonomy and ‘voice.’ Engineering loss of voice happened before in a government agency under significant financial strain. Vaughan studied the institutional arrangements within NASA in the 1980s and found how its engineers were

‘servants of power:’ carriers of a belief system that caters to dominant industrial … interests. The argument goes as follows: Located in and dependent on organizations whose survival is linked to the economies of technological production and to rationalized administrative procedures, the engineering worldview includes a preoccupation with (1) cost and efficiency, (2) conformity to rules and hierarchical authority, and (3) production goals… Engineering loyalty, job satisfaction, and identity come from the relationship with the employer, not from the profession. Engineers do not resist the organizational goals of their employers; they use their technical skills in the interest of those goals. (Vaughan, 1996, p. 205)

Vaughan’s observation suggests that calls for engineers to speak up not only mischaracterize the relationship between them and their organization—whether private or government—but that these calls are misguided. It isn’t as if engineers resist goals that arise at the level of an organization in interaction with its environment. They make them, or see them as, their *own* goals. These are no longer decisions and trade-offs made by the organization, but problems proudly owned by individuals or teams of engineers. It is an insidious but desired delegation, an internalization of external pressure facilitated by Vaughan’s “loyalty, job satisfaction and identity,” by engineers’ pride of workmanship (Deming, 1982), demonstrating that they can do more with less, that they can find solutions where others have given up. It might render calls for engineers to ‘speak up’ slightly ridiculous. Because whom are they speaking up *to*?

Outrage about an engineer not speaking up in hindsight, can lead some to make demands and call for leverage that not only insult the professional ethic and commitment of engineers in general, but that actively work against a climate of psychological safety inside the organization that employs them:

once support is available to counter loss of one’s career from speaking out about dangerous technologies, engineering societies need to require engineers to act in accordance with the prevent-harm ethic. This requirement must include both training to inculcate the prevent-harm ethic and sanctions—up to losing one’s license—when the ethic is violated. (Feldman, 2004, p. 714)

Feldman’s is not just a call for ‘safety heroes’ (Reason, 2008), it is a threat of vilification of those who aren’t. But thinking about the problem this way is more than a mischaracterization. It is a capitulation. It is an admission that the system of prospective accountability—on which the entire role of a regulator and engineering oversight is premised—has already failed. An engineer employed by a regulator for the explicit purpose of evaluating, assessing, checking and assuring the engineering of a manufacturer’s software or system, should not have to be told what a ‘test’ is, or rely on whistleblower protection, or on individual ethical heroics to probe further and be heard when something is uncovered. She or he should not have to be threatened with losing professional certification to feel compelled to look further and raise her or his voice. If that is what the field has come down to, then something much deeper, and wider, is amiss. Which, of course, is the argument made by some: a steady decades-long drift into disaster of extractive capitalism itself (Albert, 1993; Saull, 2015; Tkakic, 2019).

## The Rebuke

Guilt arising from moral injury can lead to (self-)destructive tendencies (Jamieson et al., 2020). However, for the FAA safety engineer, it led to repentance, and growth through an expression of constructive anger. His rebuke, or principled moral outrage (Rushton, 2013), led him to review the disaster and the wider conditions around it, as well as expressing compassion and empathy. These, too, are hallmarks of principled moral outrage. Let’s turn to the context so that we might better understand the reach of the rebuke.

In 2004, not long after Boeing had relocated its headquarters to Chicago from Seattle, the then COO remarked that “When people say I changed the culture at Boeing, that was the intent, so it’s run like a business rather than a great engineering firm… people want to invest in a company because they want to make money” (Defazio & Larsen, 2020, p. 34). By the time the 737 MAX was launched, Boeing—by the deliberate design of its leaders—may no longer really have been an engineering company. Where there had been twenty engineers on a part of a similar project before, there was only one such engineer for the 737 MAX. In 2014 the FAA accepted Boeing’s initial certification basis for the 737 MAX. Boeing’s stock price had been rising for a year as MAX orders rolled in. Additional sales with record profits were predicted. The company decided that from 2014 on,

a significant portion of our named executive officers’ long-term incentive compensation will be tied to Boeing’s total shareholder return as compared to a group of 24 peer companies. (Lazonick & Sakinc, 2019, p. 2)

Shareholder value and shareholder returns became a priority. As some have observed, it offered a bounty to those who had a lot of shares to begin with—including the company’s senior management. Yet the connection between rewards in shares and product safety problems was identified around the same time:

Stock options are thought to align the interests of CEOs and shareholders, but scholars have shown that options sometimes lead to outcomes that run counter to what they are meant to achieve. Building on this research, we argue that options promote a lack of caution in CEOs that manifests in a higher incidence of product safety problems. (Wowak et al., 2015, p. 1082)

Extractive financial pressure and engineering trade-offs became biased in a direction of maximal grandfathering, reduced need for oversight, less manpower allocated, accelerated approvals, no additional training requirements (Denning, 2013; Imberman, 2001). Subsequent research suggested (but could of course not prove) that with less of a singular focus on stock price during these years, resources could have been available for the safe design and development, including adequate testing and safety analyses (Lazonick & Sakinc, 2019). Nudging tradeoffs away from extractive tendencies requires a change in leadership posture—from corporate warrior to guardian spirit (Baker, 2020; Blumberg et al., 2022), listening to staff and customers, and committing not just to vague aspirations of accountability and transparency (Woodhead, 2022), but to the testable specificities of honesty and truth-telling. Repentance involves a commitment to tell the truth despite its costs. Language used both reflects and creates culture—particularly around honesty (Schauer & Zeckhauser, 2007). This means resisting concealing truth, not being fully liberal with the truth, or linguistic contortions that do not outrightly lie, but do deceive (The United States Department of Justice, 2021). To create a culture where truth is valued, it is important to actually use the word ‘truth,’ to nurture “a talent for speaking differently, rather than for arguing well, [as] the chief instrument of cultural change” (Rorty, 1989, p. 7). Accountability, if that word must be used, should be of the forward-looking kind (Sharpe, 2003): deeply connected to roles and responsibilities and focused on what needs to be done to set people inside the organization, and those tasked with its oversight, up for success.

The rebuke of the FAA safety engineer, though, goes further, reading less like the critical scrutiny of a specific act, situation or person and more like an impeachment of the political-economic and societal arrangements that enabled the disaster to incubate at the nexus between Wall Street, the regulator and the manufacturer. Because the irony is: the aircraft in question was going to be a huge commercial success—*no matter what*. And yet ever more resources were sucked from the project through extractive shareholder-capitalist arrangements (e.g. share buybacks) in which major shareholders are also the chief internal decision-makers of the company. It has made proud organizations drift into failure before (Mandis, 2013; McLean & Elkind, 2004). Going beyond its utility as personal repentance, the rebuke in the case described here perhaps serves as a political statement about ideological fandoms: deregulation, free markets and the sanctity of share price on the one hand, versus meaningful government involvement, engineering professionalism and old-fashioned metrics like earnings and operating margins on the other. The rebuke’s real subject is indignation over the kind of corporatization, deregulation and financialization that makes some shareholders wildly rich while underprioritizing engineering norms, expertise and technical know-how. It is a principled moral outrage that inspired the safety engineer to step up in the first place—in his own quoted words ‘enraged by sinful greed’ while ‘the righteous perish’ (Gates, 2021, p. 13).
